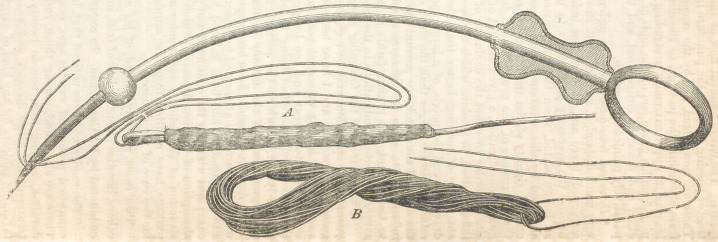# Report on Practical Surgery

**Published:** 1858-07

**Authors:** Edward Hartshorne

**Affiliations:** One of the Surgeons of Wills’ Hospital, Philadelphia


### REPORT ON PRACTICAL SURGERY FOR THE YEAR 1857.

### PART II.

By EDWARD HARTSHORNE, M.D., one of the Surgeons of Wills' Hospital, Philadelphia.

*Extirpation of Parotid.* J. M. WARREN, M.D. (Boston Med. and Surg. Journal, May 14, 1857, p. 290.)—In a paper on *Tumors in the Parotid Region*, Dr. Warren states his experience, as to an important matter, in the following paragraphs:—

"As to the practical question which is often raised, whether the gland can be removed without the ligature of the carotid, the result of my experience is this. The parotid gland has been removed by me in six instances, which are given below; three for scirrhous disease, one for erectile tissue, one for melanosis, and one for hypertrophy; in none of these was the great artery tied. The experiment of dissecting out the parotid gland in the dead subject has been frequently made by me, and with a little care this can be done in most instances, leaving the great vessels behind, although sometimes a small backward-projecting bit of the gland is left, and this has been observed to escape disease. But in scirrhous affections, where the gland undergoes a gradual induration, the vessels are frequently pushed backward, as they were in one or two of the cases here given. The above observation is confirmed by my friend and colleague at the hospital, Dr. Gay, who made similar dissections on the dead body to ascertain this point.

"In a case mentioned by Dr. J. C. Warren the carotid was cut at the end of the operation, and the jet of blood struck the wall. The vessel was secured, the carotid being compressed below, and the patient did well. In a second case for the removal of a scirrhous parotid, in which I assisted Dr. Warren, the carotid was divided and tied. Three days after, as the patient was straining at stool, the vessel gave way, and the blood struck the ceiling. He almost at once fainted, and the friends were fortunately sufficiently cool to place a sponge in the wound, and to check the flow partially. I was called, and at once cut down upon the carotid in the neck, tied it, and stopped the further effusion of blood. Bérard, in his monograph on this subject, mentions many instances of removal of this gland without ligature of the carotid."

The paper is completed, in illustration, with the history of the six cases above alluded to.

*Excision of the Tonsils.* From the Records of the Boston Society for Medical Improvement. F. E. OLIVER, M.D., Secretary. (Boston Med. and Surg. Journ., May, 1857, p. 238.)

"DR. J. MASON WARREN remarked that he had lately removed the tonsils from a child, in whom, in addition to the ordinary symptoms of obstruction to the breathing, and alteration of the voice, was produced a most remarkable spasmodic cough resembling the barking of a dog.

"Dr. W. said he would take this opportunity to speak of the result of his experience in the operation for excision of the tonsils. Some years since (1839) he had read before this Society some remarks on enlargement of the tonsils, attended by certain deformities of the chest, and the result of twenty cases was given in which the operation had been performed. This was published afterwards in the *Philadelphia Medical Examiner.* More lately, he had given the result of two hundred cases which required operation, and at present his experience would reach to above five hundred instances in which the tonsils required to be removed. These cases had not been taken indiscriminately, but the operation was only performed where the symptoms were more or less urgent, and other remedies had failed in affording relief; causing deformity of the chest, difficulty of breathing, choking at night, unnatural and offensive discharge from the mouth and nasal passages. Many of these cases were brought from a distance, on account of the importance and severity of the disease.

"In none of these cases had he ever seen any fatal acccident occur, or had reason to regret the operation. In but two cases, and those not in his own practice, but where he had been called in after the operation, had he seen any serious hemorrhage; both these cases did well. In almost all of them the symptoms were at once relieved, the patient was able to take his food with comfort, to sleep better, and exchanged a pallid and depressed aspect, for a healthy and animated appearance. To the rapidity with which some of them had gained flesh, as soon as a proper amount of oxygen was allowed to penetrate to the lungs, many of the gentlemen present would bear witness. He could conscientiously say that he knew no minor operation in surgery that afforded greater relief and more satisfactory results than the one under consideration.

"In answer to the question whether the tonsils were ever reproduced, requiring a repetition of the operation, Dr. W. said that in four or five instances only had he been obliged to repeat the operation. The whole of the tonsil never is or ought to be removed. When the enlargement is very great and irregular, it sometimes extends down the throat with a broad base, and it is not possible to embrace at once in the instrument as much of the tonsil as it would be de sirable to remove, and the apex only is excised. The consequence is, that the lower portion afterwards rises up and comes into view, causing obstruction, and requiring another operation. These cases were, however, very exceptional.

"The instrument that Dr. Warren had always used was the guillotine instrument, introduced into practice by Dr. J. C. Warren—made perfectly simple, without any needle or spring to seize or drag out the part to be removed. The thick mucus of the fauces causes the portion to be cut off to stick to the instrument, so that it seldom escapes into the throat. The objection to those instruments which cut by pulling the knife out is, that they require to be kept constantly sharp, otherwise the tonsil may be dragged or torn out. The guillotine instrument does not require this, in fact it is better dull, causing less hemorrhage, and possibly a subsequent greater destruction of that part which remains. His own instrument had been to the instrument-maker's but once or twice for the last fifteen years. It was kept bright and in good order by not putting the blades together except when used."

Three cases were then referred to, by different members, in which death from suffocation was due to enlarged tonsils, and a third case in which convulsions were attributed to the same cause.

*Case of Spasm of the Glottis, successfully treated by Laryngotomy.* DAVID CRARY, M.D. (Virginia Med. Journal, Feb. 1857, p. 123.)—Dr. C. was hastily summoned, November, 1844, to a boy, two and a half years old, who was "choked." He found the glottis completely closed. In a few seconds more the respiration ceased; the face became livid, and all the symptoms of asphyxia supervened. The friends, as well as himself, supposed that the child was dead.

"The thought occurred to me at once, that there could be no harm in opening the trachea, to give the little sufferer the only possible chance for his life. I took him from his father's arms, and placed him across my knees, and, with a common lancet, aimed to make a longitudinal incision through the thyroid cartilage, as near the median line as possible. Owing to the bad position in which I held the patient, and the want of assistants, for whom I had no time to wait, I made the opening a little to one side.

"For the first four or five seconds, it seemed as though the operation would be of no avail; but in a moment more, air and blood were expelled through the incision, and the breathing was presently re-established; so that very soon, when the hemorrhage ceased and the lips of the wound were kept apart, respiration was carried on as freely through the artificial opening as it formerly was through the glottis.

"In the course of an hour, the spasm of the glottis subsided, and the little fellow would breathe first through the mouth, and then through the wound, to his great amusement.

"I allowed the incision to remain open until the next morning, when, all symptoms of dyspnœa having disappeared, I drew the edges of the wound together with adhesive strips. On the second day after the operation, the child played about his room, as though nothing unusual had occurred. At the end of a week, the parts had completely healed, by first intention, and without an unpleasant symptom.

"When I had time to inquire into the condition of the boy previous to the paroxysm in which I first saw him, I learned that he had been put to bed on the evening referred to, in perfect health, as the parents supposed. The distressed breathing was not noticed until a few minutes before my arrival."

*Tracheotomy for the removal of a Pebble from the Trachea.* DR. SLATER. From the Records of the Boston Society for Medical Improvement. F. E. Oliver, M.D., Secretary. (Boston Med. and Surg. Journ., March 1857, p. 82.)—About eight weeks previously, while jumping in a fright to a piazza, the patient, a little boy, was suddenly seized with violent coughing and symptoms of strangulation.

"A woman who was near at hand, and to whom he made signs indicating that something was in his throat, put her finger into his fauces in order to relieve him, and said that she felt the pebble, and while attempting to remove it, it slipped from her fingers. Almost immediately the violent symptoms subsided, and the boy ran home. From this time the child had occasionally had violent fits of coughing. This cough was very peculiar; being short, suffocative, and slightly stridulous. During the day, for the most part, if kept quiet, he had very little cough, and his breathing was quite easy and natural; but on making any exertion, as in running or going up and down stairs rapidly, coughing was excited, and his breathing rendered so difficult and laborious that it could be heard in almost every part of the house. The fits of violent coughing seldom occurred during the day, unless there was extra exertion. At night, however, after going to bed, the fits of coughing and difficulty of breathing were particularly severe and distressing.

"While the boy was quiet, it was noticed that his expiration was more difficult than his inspiration. On applying the ear to the chest, either front or back, light sonorous and sibilant sounds were heard. They were most distinct when the ear was applied to the acromial region of the right side, either back or front. After exertion these sounds were louder. The whistling sound would disappear for a short time after a fit of coughing, and then return.

"Dr. S. gave it as his opinion, that there was a foreign body in the trachea or one of the bronchi, and advised an operation for its removal."

This being objected to, an emetic was administered to no purpose, and the child was not seen by Dr. S. until two months had elapsed. During this interval,

"Besides the usual symptoms, he had passed through a mild attack of lung fever. The paroxysms of violent coughing became more and more frequent, more distressing, and more prolonged, continuing, for the last few nights, from three to four hours. He complained more frequently of suffering an intense fixed pain in the upper part of the trachea, commencing with the cough, and lasting for some time after the fit was over, but gradually subsiding into greater or less continuous soreness. His appetite was failing and his flesh emaciating. The father was again urged to submit his child to an operation, as affording the only chance to save him."

This was finally agreed to, and performed Feb. 5th, 1856, at 12 o'clock, M.

"In the morning of the day of the operation, during a paroxysm of coughing, the stone was heard forcibly driven to the upper part of the trachea two or three times, conveying the idea of a light clicking sound at the upper part, and a duller sound at the lower part of the trachea. The boy complained of the usual pain at the upper part of the trachea after the fit of coughing was over. This freedom of the stone in the trachea gave reason to hope that there would be little difficulty in finding it; in short, that it was altogether probable it would be expelled from the trachea the moment a sufficient opening should be made.

"The operation was performed in the presence of Drs. Coale, Hodges, and Coolidge. The patient having been placed upon the table and etherized, he was put in position for the operation. An incision was made exactly in the median line, about two and a half inches long. The skin, fascia and fat being divided, the sterno-hyoid muscles separated, the loose cellular texture and veins being removed from the front of the trachea, and the thyroid gland pushed out of the way, the trachea being stretched and fixed by the assistants, and the wound perfectly cleansed of blood, a knife was inserted into the trachea at the lower part of the wound, and three or four rings divided by carrying the knife upward. A moment after the opening was made, the stone was thrown into the opening by a short and sudden cough, and before it could be seized by the forceps, another short cough threw it out upon the napkin. A curved conical silver tube was introduced into the trachea, to prevent the entrance of blood. As soon as all danger of this had ceased, the tube was removed, and the wound dressed with folds of linen cloth moistened with water. These were changed as often as they became dry. In the evening the wound was closed with adhesive plasters. The night was passed more quietly and comfortably, and with more sleep, than the boy had had during any one night for four months. The stone was smooth, and about the size and shape of a Lima bean. After the operation the wound healed rapidly, the patient improved in health and strength, and is now, Feb. 14th, entirely recovered."

*Successful Tracheotomy for Piece of Beef Bone in the Larynx.* DR. C. JOHNSTON, of Baltimore. From Extracts from the Transactions of the Baltimore Pathological Society, by W. Chew Van Bibber, M.D. (Virginia Med. Journ., April, 1857, p. 269.)

"The subject, a boy of six and a half years, in the enjoyment of perfect health, while partaking of shin soup at dinner, was observed suddenly to make a hissing noise in his breathing, which was performed with difficulty and spasmodically—and presently his rosy color changing to a dingy or leaden hue, the child's parents became alarmed for his safety, and procured the attendance of a physician."

Dr. Brewer was subsequently called, and invited the aid of Dr. Johnston.

"Dr. Johnston verified Dr. Brewer's diagnosis in every particular. The boy was suffering exceedingly from an obstacle, supposed to be a morsel of bone, lodged in the larynx, while additional embarrassment appeared to exist in the bronchia. Emetics, the feather, and shaking had already proved ineffectual; wherefore the operation already named was suggested, and it was approved of by the parents, who perceived the danger of a 'masterly inactivity.'

"No chloroform was given, of course. A strong man, sitting astride a narrow table, supported the child seated in front, and restrained his motions. An incision through the skin was now made in the median line, from the thyroid cartilage downward, for an inch and a quarter. Several prominent, turgid veins were next held carefully aside by Dr. Brewer. Dr. J. then seized the cricoid cartilage with a tenaculum, point upward, punctured the trachea with a scalpel guided by the tenaculum, and inserting a probe-pointed bistoury into the wound, cut downward, while the larynx was drawn toward the chin.

"The patient instantly was seized with a paroxysm of coughing, and discharged through the artificial aperture two long, tough masses of mucus that had doubtless been lying in the great bronchia, while the hemorrhage, which had been considerable, almost entirely subsided as respiration became free and more tranquil.

"Now that the danger of suffocation was averted, a short pause was allowed, that the child might be tranquilized. By probing the larynx, a grating substance could be felt, but the forceps could not reach it, and it was found impossible to dislodge it from the glottis by means of an elastic catheter inclosing a bent wire introduced into the mouth.

"A long silver probe was at this juncture bent into a wide curve, and a strong double silk string passed through the eye, and attached firmly below to a bit of sponge. To facilitate the manœuvre, as the neck was short, the cricoid ring was divided. Then the probe was passed upward into the mouth, and drawn out; the sponge engaged in the larynx, and finally traction upon the string over a finger passed between the fauces brought out the sponge, and with it the 'front of all offending.' It was a piece of cancellated beef bone three-fourths of an inch in length, one-half of an inch in width, and one-fourth of an inch in thickness, and is supposed to have been situated partly between the vocal cords and partly above them.

"Immediate relief followed the extraction, and the cessation of all manipulation except cleansing the wound, which was not closed; and the boy was left breathing through it as well as through the mouth.

"During the night there was fever and slight delirium; pulse 180, and respiration 60; but in the morning the pulse had fallen to 160, and respiration to 52, under the judicious management of Dr. Brewer. Digitalis, ipecac. and soda were exhibited, ice applied to the head, and iced drinks administered, and a daily improvement became evident.

"To-day, 24th December, (8th day,) the wound, which was granulating finely, was closed without inconvenience. Pulse 95; respiration 32; condition excellent—well."

*Removal of a Foreign Body from the Trachea by Tracheotomy.* R. E. HAUGHTON, M.D., Richmond, Ind. (Cincinnati Med. Observer for Nov. 1857.)—The accident occurred on the evening of Aug. 15th, 1856, in eating a melon, one of the seeds being drawn from the table with the juice of the melon.

"The patient was a little girl, about four years of age, just tall enough to reach the table, and applying her mouth, thus sucked the seed into the trachea. There was some spasmodic coughing at first, but after a time, the respiration was not materially disturbed. The next morning, however, there was some wheezing and difficulty of breathing, the sound of the seed striking against the sides of the trachea, as the air passed in and out in the process of respiration."

The parents of the child were prepared for an operation as the only resource, in case of urgent symptoms supervening.

"The next day arrived, being Monday the 17th of August, and the prospects and condition of the patient no better. In the evening, during a paroxysm of coughing, the seed was thrown upward, and lodged in or about the chink of the glottis, when the symptoms became rapidly worse, and Dr. Plummer, Dr. Butler (my partner,) and myself were hastily summoned to the case.

"The incision was made down to the trachea, then three or four rings were divided, freely admitting the air. There was no bleeding of importance, but the seed was not expelled. The color soon returned, and though nearly pulseless, soon rallied a little, but being late in the day and she being so much exhausted, she was quietly laid in bed and watched through the night, a silver tube having been immediately inserted through the wound for the purpose of respiration. Next morning she was as comfortable as could have been expected, and we proceeded to find the locality of the seed, and if possible remove it. The seed was lodged or impacted somewhere above the opening into the trachea, and could not be readily found. It was thought to be in the chink of the glottis, or near by, or might have been found in one of the ventricles of the larynx, but as this was uncertain, we first passed a small bougie upward to the chink of the glottis, but could go no further. This was done with the view possibly of dislodging the seed, but as yet it was not found. The next effort Dr. Butler took a pair of curved forceps of the pocket case, and passing it upward slowly through the opening in the trachea, seized the seed and brought it to the external margin of the tracheal wound, and a struggle of the patient dislodged it from the forceps as it passed down below the wound in the trachea. We then supposed it would be expelled by coughing, and she was again laid in her bed, and soon after in an effort at coughing the seed was expelled, and caught in my hand as it was thrown out. The next steps were to combat inflammation, which was done through that day and night, by the use of nitrous powders, and the tinct. verat. viride, which exerted a most happy influence. I watched her through that night, and she seemed to grow more and more feeble, her pulse rose to 140 beats per minute, and all the members of her family had consigned her to death. I gave at intervals the tinct. verat. viride, till her pulse was reducd to 60 beats per minute, and more volume and softness. From that hour she rallied and continued to improve, and the next morning I told her father she was better, and improving; he was incredulous, and would hardly believe, even through that day, that she improved. She did not breathe through the natural passages till the Thursday following, when the tube was removed and the opening closed. She improved rapidly, and the following Sabbath, one week from the time I first saw her, her parents took her on a visit to friends in the country."

*Removal of a Piece of Tin from the Windpipe by Laryngo-Tracheotomy.* PAUL F. EVE, M.D. (Nashville Journal of Med. and Surg., Sept. 1857, p. 234.)—A negro boy, aged three years, had, two days previously, while attempting to whistle with a small strip of *tin* in his mouth, fallen backward and inhaled it into his windpipe. The usual embarrassment to respiration, ronchus, alteration of voice, and in this case some slight hemorrhage, immediately followed. Not having been relieved by "an emetic, inversion of the body, &c.," he was sent to Dr. E. on the 11th of June. On the 13th "the larynx and the trachea were opened, and diligent search made for the foreign substance." "The larynx was twice wiped out by sponges carried through the wound and brought out of the mouth, and the trachea and bronchi carefully and cautiously explored with probes and long forceps, but nothing special detected." The operation was unusually difficult and tedious; the child being fat, the hemorrhage abundant, and the effusion of mucus so extensive, that, counting the time consumed in arresting the hemorrhage, and using the anæsthetic (equal parts of ether and chloroform) with extra precaution, he was nearly two hours upon the operating table, and yet no one could discover the foreign body.

The piece of tin (measuring $\frac{7}{10}$ths of an inch in length and $\frac{3}{20}$ths in width, with smooth surface and sharp angles and edges) was ejected through the wound, during a paroxysm of a cough, on the 15th, about two days after the operation. The slight inflammatory action, for which a mild antiphlogistic treatment had been prescribed, soon subsided. The opening was gradually closed with adhesive plaster. On the eighth day after the operation the patient was up and dressed, and the wound was healed by granulation. On the ninth day his breathing was again nearly natural, and on the tenth he returned home well.

*Radical Treatment of Hydrocele of the Tunica Vaginalis.* GEORGE FRIES, M.D., Cincinnati. (Western Lancet, Feb. 1857, p. 81.)—Dr. F. objects to all the usual modes of operating in this disease, including injections, and prefers the tent alone. His plan "is to firmly grasp the posterior part of the tumor, and with a common scalpel make a free incision of an inch or more into the lower and anterior portion of the sac."

"As soon as the fluid has escaped, I pass, by the use of a small spatula, a piece of linen a half or three-fourths of an inch in width, over, and spread it upon each side of the testicle, leaving the ends of the tent to project from the wound below. These tents are permitted to remain in their place from twenty-four to forty-eight hours, according to the susceptibilities of the part. Within this time, the necessary degree of inflammation being excited, they are removed. The wound is to be kept open below for the free discharge of serous, purulent, or other matter that may form. Soon as the operation is completed, I direct that the scrotum be continually bathed with a mixture of equal parts of whisky and water. To derive the full advantage of this, it is necessary to apply it constantly and see that the cloths about the parts are so light as not to interfere with the process of evaporation.

"Occasionally it has been necessary to use saline cathartics and tart. emetic, to allay febrile excitement. Sometimes an opiate has been required to allay pain and produce sleep, but in the main, I may say my patients needed but little medication.

"I have now operated in this way," says Dr. F., "in more than forty cases, sometimes on the infant a few months old, sometimes on the old man of seventy, and in several instances where the cure had been previously attempted by injection, and I have not failed in a single instance, nor have I witnessed any untoward effects but in two cases, one of erysipelas, the other from hydro-sarcocele, in which I had subsequently to remove the testicle."

DR. BYFORD, of Evansville, Ind., recommends (*Cincinnati Med. Observer*, Feb. 1857, p. 73) puncture with a lancet, and continued evacuation, as a still milder and simpler plan, which had succeeded with him in three trials. He considers two circumstances essential to success in his operation:—

"1st. That the puncture should be made in the most dependent part; and 2d, that adhesion between the lips of the wound should be broken up as often as formed, until all the fluid is evacuated and adhesive inflammation is set up. We thus establish a kind of fistule, which admits for the time a slow evacuation of the serum, and probably also allows a sufficient ingress of the air to cause a mild degree of inflammation. In all three of the cases the puncture was small and the evacuation at the first sitting was not complete. It continued dribbling away by degrees for the first three days."

*Fractures of the Neck of the Scapula.* L. A. DUGAS, M.D., Professor of Surgery in the Medical College of Georgia. (Southern Med. and Surg. Journ., June, 1857, p. 336.)—In a paper on Fractures of the Scapula, read before the Medical Society of the State of Georgia, April, 1857, we find two cases of fracture of the neck of this bone:—

"CASE I.—On the 7th of October, 1853, a stout negro man, about nineteen years of age, called Ambrose, and belonging to Mr. Avery, of Columbia County, Ga., was sent to me with a note from my friend Dr. H. R. Casey, who had seen the case. It seems that three weeks previously, while at work in the field, a limb fell from a tree upon the left shoulder of this man. The blow was very severe, and, upon recovering from the shock, the man found that he had entirely lost the use of his arm, but suffered excruciating pain in the shoulder, axilla, and even to the ends of his fingers. The Doctor saw him a few hours after the accident, and found him still suffering intensely and unable to move any portion of the limb, not even the fingers. No arterial pulsation whatever could be felt at the wrist, the limb was rather cool, but sensibility was not destroyed in it, for the patient would feel when pinched. There were no symptoms of concussion of the brain nor of any lesion about the head. The shoulder alone had been stricken, and this was very much swollen. Opiates were freely administered to relieve the pain, and the limb was placed in a sling."

Dr. D. diagnosticated fracture of the neck of the scapula still existing, "and thought that the paralysis was induced by injury to the axillary nerves and vessels which were jammed against the ribs by the head of the humerus, when the blow was received." In a letter to Dr. C. he briefly enumerates the symptoms observed, and recommends a plan of treatment, in the following words:—

"Evident depression of the head of the humerus below the acromion; the head of the humerus rotates under the finger continuously with the lower end of the bone, and without crepitation; the depression of the head of the humerus is reduced by pressing up the elbow; crepitation very audible, and easily felt by placing the left hand upon the shoulder, while with the right hand you seize the elbow and work it freely, so as to force the shoulder up and down. No crepitus can be induced by acting upon the different parts of the *body* of the scapula, nor upon the acromion, nor upon the clavicle. By placing your ear, with or without the stethoscope, upon the scapula, the crepitus is very loud. Now, as to the suspended circulation and paralysis, I find no pulsation in the arteries, not even as high as the axilla, although the artery can be felt with its accompanying nerve on the inside of the biceps. The limb is cold, but especially so, below the elbow. Circulation in the veins evident, but slow. The limb is insensible below the elbow, and partially so above. The ulnar nerve may be compressed behind the elbow without sensation.

"The patient says that he suffered dreadfully at first, and that the whole limb down to the ends of his fingers was much swollen, as well as that side of the chest, for some time after the injury; and that loss of motion was immediately induced. From these facts, I think myself warranted in the inference above indicated, and also in the belief that the vessels have suffered so much from the contusion as to obliterate the axillary artery.

"It is now three weeks since the accident—what is to be done? Suspend the elbow with a handkerchief sling, such as I advise in fractures of the clavicle, so as to keep the fractured edges in contact, and to relieve the axillary nerves from compression. Give electric shocks daily to the limb, passing the fluid from the back of the neck down to the fingers. This stimulation of the nerves may possibly be useful. Let the man take exercise to brace the system."

"*Remarks.*—I should here observe," says Dr. D., "that when I saw the patient I could not feel the coracoid process with sufficient distinctness to act upon it as advised by Sir A. Cooper. Yet the whole chain of symptoms was sufficiently characteristic to leave no doubt as to the true nature of the case. The head of the humerus was depressed to such a degree, as to rest upon the axillary plexus, but could be easily forced up into its proper position; it rotated *continuously* with the shaft of the bone, and without crepitation; crepitation was easily felt by forcing the elbow up and down so as to bring the fragments against each other. Neither the clavicle, the acromion, nor any portion of the *body* of the scapula was broken. The seat of fracture could only be in the surgical neck of the scapula.

"The lesion of the axillary artery and nerves here noted deserves especial attention, for I am not aware that it has been mentioned by any author in connection with this accident. It is true, that some of them allude to the disability sometimes experienced in the use of the limb, and which may even lead to loss of motion in neglected cases; but, nowhere do I find any intimation of the sudden production of paralysis, nor of the obliteration of the artery so remarkable in this case, and so evidently caused by the immediate injury done to the nerves and blood-vessels of the axilla."

"CASE II.—In December last, (1856,) Mr. R. W. Daniel, of Jefferson County, in this State, brought to me a negro man between fifty and sixty years of age, who, in the month of March previous, while felling trees, was stricken down by a limb. The blow was principally sustained by the right shoulder, and he immediately lost the use of his arm and fingers. The physician who saw him reports that no pulse could be felt at the wrist at his first visit. My examination of the case, nine months after the accident, revealed the following state of things:—

"The clavicle had been broken at about two and a half inches from its acromial extremity, but the fracture was united, the sternal fragment overlapping the other. The acromion process had also been broken at its junction with the spine of the scapula, and an uneven union had taken place. The connection between the acromion and the clavicle appeared to have sustained some injury, as it was enlarged and uneven. The shoulder drooped so much as to resemble very much at first sight a dislocation downward. The deltoid was flattened, and the head of the humerus could not be found in its proper place, unless forced up by acting upon the elbow, by which means it could be readily replaced, but would again immediately fall upon releasing the elbow, and permitting the limb to hang down. The coracoid process evidently followed the upward and downward movements of the humerus. No crepitus could be detected by rotating the humerus when hanging down, but it became very audible when the elbow was forced up and then moved in different directions. The elbow could be placed against the chest while the fingers rested upon the sound shoulder, without any difficulty, so as to establish conclusively that there was no dislocation of the humerus, according to the principle I have established for some years back. (See *Southern Med. and Surg. Journ.* for 1856, p. 131.)

"The patient still suffered almost continual pain along the arm down to the fingers. He represented his sufferings as sometimes excruciating. These were somewhat relieved by forcing up the head of the humerus. He was entirely unable to move any muscle from the shoulder down, and he said that his limb felt benumbed, especially from the elbow to his fingers. He could, however, feel when pinched at any point of the surface. No pulsation whatever could be felt in any of the arteries of the limb, and the temperature of the skin was lower than that of the other arm. The pectoralis major, the muscles of the scapula, those of the shoulder, and indeed of the whole limb, were very much atrophied. The man's general health had suffered from long-continued pain and want of accustomed active exercise.

"The only prescription made in this case was, that the humerus should be well forced up and sustained in this position by means of a sling bandage carried beneath the elbow and well secured.

"Dr. James Bell, who lives in the neighborhood, informs me in a letter, (dated 18th March, 1857,) that the patient has been using the sling since I advised it, that the shoulder appears to be a little fuller, but that he still has no use of his limb, and occasionally suffers extreme pain in the wrist. His fingers are dwindling away and becoming stiff.

"*Remarks.*—It will be observed that, with the exception of the fractures of the clavicle and acromion, this case presents a striking analogy to the one just preceding. In both cases the injury resulted from the fall of a tree, in both instances the blow was followed immediately by paralysis and cessation of circulation in the arterial trunks, and in both the injury to the nerves and blood-vessels has persisted. Both have derived some relief by the support given to the limb, but they still suffer more or less. In neither case was a retentive bandage applied soon enough to promise union of the fragments, and none has taken place. Both being ignorant and heedless negroes, have doubtless been more or less remiss, in proper attentions to the application of the bandage, and this may account in some measure for the persistence of pain. Would a timely application of suitable bandages have allowed the nerves and vessels to recover from the severe injury inflicted upon them? The question cannot be satisfactorily answered without additional facts and observations.

"The deplorable condition of these men, who have not only lost the use of an arm, but are also subject to harassing pains, which may continue indefinitely, would seem to demand at our hands some measure of relief. Unable to think of anything better, the propriety of amputation has presented itself to my mind as perhaps justifiable under the circumstances.

"By removing the weight of the limb which is continually pressing upon and chafing the nerves, it is probable that the pains with which these patients are annoyed would cease. The usefulness of the limb being irrecoverably lost, the only objection to amputation, since it may be done without pain under the influence of anæsthesia, would be its danger to life. This danger would of course be greater in amputation at the shoulder-joint, than if it were performed at the insertion of the deltoid muscle, and the latter would probably answer the indication. Amputations in the upper portion of the arm are so rarely fatal in this section of country that the patient might well take the risk for the relief, especially when we consider that the change from a life of laborious exercise to one of even painless inactivity is in itself not without danger."

*Two Cases of Recovery from Fracture of the Spine, with Remarks on this Fracture.* FREDERICK D. LENTE, M.D., Surgeon to the West Point Foundry. (American Journal of the Medical Sciences, Oct. 1857, p. 361.)

"CASE I.—Barney McGuire, aged about forty, of ordinary health, a 'helper' in the foundry, fell a distance of twelve or fifteen feet while at work on the 9th July, 1853, striking on his back. I did not see him until about four hours after the accident, when I found him in the following condition—he was lying on his back in bed, his countenance pale and indicating great anxiety and prostration, intellect perfect; pulse rather feeble; stimulants had been already administered; was complaining of severe pain in the dorsal region, and 'numbness' in the lower extremities. Had not passed his water since the accident. There was complete paralysis of motion in the left limb, and almost complete in the right, being just able, with great difficulty, to flex the knee slightly, and to 'stir the toes.'

"Upon examining the back, the only point at which he complained of pain, I found a considerable projection at the situation of the lower dorsal vertebræ; upon pressing upon which, patient complained of great pain and soreness, so that it was difficult to make out whether it was due to extravasation, or to injury of the bones. He was immediately cupped over this point, and directed to be kept as quiet as possible in bed. After the cupping, a little power of motion in the right leg was gained, and he complained of a pricking sensation in both limbs. Paralysis of sensation was evidently less. His urine was also drawn off."

We have no room, unfortunately, for the details of the progress and treatment of this case. There is nothing unusual in the symptoms, except, perhaps, an irritability of the bladder, which subjected the patient to great suffering when the urine was allowed to remain longer than six hours undrawn. Sensibility, power of urinating, and a limited amount of motive power, gradually returned under the use of strychnia internally, and the application of electro-magnetism locally; the effect of the strychnia being very decided.

"CASE II.—This patient, a strong, healthy young man, aged about twenty, an apprentice to a tinman, was stepping from the roof of a house to a narrow scaffolding below, when he lost his balance; finding that he was about to pitch head foremost to the ground, a distance of about thirty feet, he sprang forward, and alighted first on his feet, and then received the force of the concussion on his seat. The ground was firmly packed clay and gravel, yet the force was so great that his buttock made a distinct depression. He was taken up pale, exceedingly prostrated, and almost senseless; he soon, however, recovered to some extent. It was found immediately that he was completely helpless below the hips, and he was taken home on a cart.

"Upon a thorough examination of the patient, after having him placed on a bed, and after reaction had set in, there was found to be complete paralysis of *motion* of both lower extremities. Paralysis of *sensation* was complete on the *right* side, from a little below the crest of the ilium downward; on the *left* side the paralysis of sensation was not so complete. Paralysis of the *bladder* complete. Upon pressing along the spine, no pain was complained of until the lumbar region was reached; at about the middle of this, the tenderness was very great, but there was no perceptible irregularity. The patient remained under my care for about two weeks, and was then removed on a litter to Orange County, where his parents reside; for the further history of the case, and its treatment, I am indebted to the kindness of his physician, Dr. J. H. Thompson, of Goshen, whose own language I shall preserve, as far as possible, in continuing the case."

This, also, we are compelled to omit; and must confine our further quotation to the first paragraph of the concluding remarks; our object being to note the fact of *recovery*, or, rather, *survival, with partial recovery*, in both cases, and the probable active remedial agent,—strychnia.

"There are several notable features in the above cases, upon which it may not be out of place briefly to remark. The fact that, in both cases, the muscular power was greatest in the limb having the least sensibility, in one no sensibility at all; the long-continued use of *nux vomica* and its alkaloid, and its undoubted efficacy in both cases, is a feature also worth noting. I believe so decided evidence of the good effect of this agent in paralysis from injury has seldom been observed or recorded. The most important point, however, is the recovery of two cases of complete fracture of the spine, whether this result be attributed to the treatment at all, or to the powers of nature, in resisting and repairing the effect of severe injury, or to both. This is a point of considerable practical importance. Complete fracture of the spinal column has almost universally been regarded by authorities, as will be seen by the references at the beginning of this article, as necessarily fatal. In consequence of this, no special efforts are usually made with a view of curing or partially curing the case, or of prolonging life until the recuperative power of the system may step in, and assist us in alleviating at least the wretched condition of the patient.

*Fracture of the Pelvis during Pregnancy.* J. WHITAKER, M.D., of Lewistown, Niagara Co., N. Y. (American Journal of the Medical Sciences, July, 1857, p. 283.)—On the 18th of December, 1856, Mrs. W., then in the seventh month of gestation, informed Dr. W. that she had fallen the day before, "slipping down a pair of steps, and striking astride the edge of an open upright barrel." She complained of excruciating pain in the left pubic region on the least motion. He found an oblique fracture of the body of the left os pubis, with but little displacement, and no lesion of the bladder or rectum, and no positive attempt at miscarriage.

"A roller bandage was applied around the pelvis, opiates were administered, and the urine drawn off for three days by means of the catheter. No bad symptoms supervened, and in six weeks the patient was able to walk about the room. On the 6th of March inst., she was delivered, after an easy labor, of a healthy female child, weighing ten pounds. The fracture was, of course, re-opened, and I was not surprised in seeing symptoms of peritonitis present themselves; these were promptly met by blood-lettings, and the exhibition afterwards of calomel and opium. The inflammation yielded kindly, and I am happy to state that at this date the patient is doing exceedingly well, and will be up in a few days."

*Fracture of the Neck of the Thigh-bone.* R. D. MUSSEY, M.D., Professor of Surgery in the Miami College, Cincinnati, Ohio. (Amer. Journal of the Medical Sciences, April, 1857, p. 299.)—The object of this paper is to describe, and illustrate with accurate engravings (nineteen in all) of the specimens, a number of cases of intra-capsular and extra-capsular fracture of the neck of the femur, in three of the former of which the author thinks that the fact of bony union is fully demonstrated. The histories of five of the cases are given in detail. The first case is one which has long been familiar to many surgeons, abroad and at home, as Dr. Mussey has been in the habit of showing the specimen, with various success as he states himself, to every distinguished authority with whom he met.

"In the year 1830, I showed this to Messrs. Roux and Amussat, and some other professional gentlemen in Paris; they regarded it as a fair specimen of bony union of intra-capsular fracture. In London, I also showed it to Mr. Lawrence, Mr. Travers, Mr. Stanley, and Dr. Hodgkin, who was then Curator of the Museum at Guy's Hospital. These gentlemen were interested with the specimen, and considered it as a satisfactory example of bony union within the capsular ligament. On my presenting it for inspection to Sir Astley Cooper, he remarked 'This bone never was broken.' I said, 'Sir Astley, please to look at the interior of the bone.' He separated the two halves, and said, 'This does look a little more like it, to be sure; but I do not think it is wholly within the capsular ligament.' It is well known that Sir A. Cooper, for some years, had taught the doctrine that bony union does not take place in the intra-capsular fracture. His views, among the surgeons of Great Britain, were extensively admitted as correct.

"At Edinburgh, I showed it to that distinguished surgeon, John Thompson, whose work on inflammation had given him extensive notoriety. On carefully inspecting it, he declared, 'upon his truth and honor,' that it had never been broken. An opinion had prevailed for some time among surgeons, that in old persons the head of the thigh-bone is liable to sink below its ordinary level, with more or less shortening of the neck; which occurs in certain morbid conditions without the aid of mechanical violence. Mr. Thompson regarded this as an instance of that kind of change.

"After returning to America, I obtained the right thigh-bone from the same skeleton. Fig. 3 [p. 301 *Amer. Journ. of Med. Sciences*, April, 1857,] gives a very correct delineation of the head, neck, and upper part of the shaft of this bone. Between this and the other specimen the difference is very striking. The neck of this exhibits the ordinary angle with the shaft, and there is no depression of the head, and no mark across the neck.

"The professional gentlemen of our country who have examined these specimens, unhesitatingly pronounce this to be a case of union by bone of intra-capsular fracture."

These histories, and Dr. Mussey's discussion of them, as well as his representations of the specimens, are so interesting that we regret our inability to quote more largely from his paper. It is the fruit of more than thirty years' experience and careful study in an extended field; and is, therefore, in addition to its practical importance, entitled to unusual scientific authority.

We have room only for one short extract which may indicate his surgical creed upon this long-vexed question. It has, at least, the advantage of the positive side, and the merit of hopefulness.

"All, then, that is required for the bony reparation of fracture, the constitutional health being good, is the undisturbed apposition of the broken surfaces. Thus, in those forms of extra-capsular fracture in which the neck of the thigh-bone is driven into and firmly impacted in the cancelous texture of the great trochanter, osseous union follows independently of callus; but when there are several fragments which are exposed to motion, callus steps in to hold them steady until the injury is repaired. When a fracture lies within the capsule of a joint, callus is not admitted there, as it would abridge or destroy the natural motions of the articulation: and the work is done by a flexible fibrous bond of union, when the broken surfaces are too far asunder, or have too much mobility for bony solidification. Is not all this as it should be, and does it not afford proof of intelligence and wisdom behind, giving direction and guidance to these processes?"

*On Intra-Capsular Fracture of the Cervix Femoris.* Inaugural Thesis. JOHN G. JOHNSON, of Mass. (N. Y. Journal of Medicine, May, 1857.)—Dr. Johnson takes the negative side of the question as to bony union of intra-capsular fracture of the femur, and devotes twenty-five pages to an elaborate and well-conducted attack upon "all the cases of supposed osseous union." The concluding paragraphs may serve to show the tone and course of his argument.

"All of them are defective in points of great importance; no one of them places the question beyond a reasonable doubt, and it should be remembered that these are the choice specimens of all Europe and America.

"There is a single point more worthy of notice; it is the extreme difficulty there appears to be, in all these specimens, of deciding whether they are intra-capsular, or only partly within. The mere fact that a surgeon of such eminence as Professor Mussey should have been misled, and should have been so deceived as to take a specimen across the Alantic, to convince Sir Astley Cooper of the possibility of ossific union of intra-capsular fractures, by whom it was conclusively shown that he was in error, is sufficient to prove the difficulty of deciding on these specimens. All such specimens should be preserved in the wet state with the capsule still attached, when there could be no doubt of their character.

"The argument may be thus summed up:—1. It is absolutely impossible to form a certain and unmistakable diagnosis of all these fractures during the life of the patient. 2. That the probabilities are all against union by bone, from lack of nourishment to the fractured parts of the head, from impossibility of perfect rest, and from synovitis. 3. The argument from the analogy of fractures of the patella, olecranon, etc., is an argument against, instead of for, osseous union. 4. Cases where every circumstance was favorable to union, if any fractures of this kind could unite, yet which failed to unite. 5. The cases given to prove this union not having proved it.

"The *treatment* of these cases is obvious. It has been shown that a positive diagnosis cannot be formed. It may be a case of those kinds which do have a bony union. The patient is entitled to the benefit of this chance. If his age and health will permit, the straight splint should be used; while, at the same time, the surgeon should protect himself, by showing the patient and his friends what an unfavorable result there may be. If the fracture is impacted, it should by all means be kept in the impacted state, and all attempts to extend the limb to its proper length should be abstained from.

"If the patient is past the prime of life, or of enfeebled constitution, then the double inclined plane splint should be used; and if the patient cannot bear that, then simply let the limb rest over a pillow."

*Ununited Fracture successfully treated.* Dr. JOSIAH CROSBY. From the Report on Surgery, by Dr. G. H. HUBBARD. (Trans. of the New Hampshire Med. Society, 1857, p. 28.)

P. A., an Irishman, aged twenty-two, of temperate habits and good constitution, fell twenty feet, and broke both bones of his left leg midway between the knee and ankle. The fracture (a transverse one) was treated as usual. At the end of the third month, when Dr. Crosby first saw him, he was walking on crutches, with both bones ununited. The leg was dressed on Roe's splint, (very much like Salisbury's,) flexed to nearly a right angle with the thigh, the thigh made fast to the splint by straps, as also the leg below the knee, and the ankle screwed round so as to make firm and constant pressure against the bottom of the foot. In four weeks the dressings were removed, and union effected.

J. D., an Irishman, aged twenty-four, in perfect health and temperate habits, suffered a comminuted fracture of the humerus, just above the insertion of the deltoid muscles; a piece of the bone, quite one-third of its diameter, and one inch in length, was split from the end of the upper fragment, and drawn an inch above the fracture. Severe inflammation followed, and eventually subsided without other untoward result than non-union of the bone. Eight months after the injury there was free motion at the seat of fracture.

Resection was performed, and the fragments fastened together with gold wire, the arm being then dressed with a gutta-percha splint fitted to the arm and encircling three-fourths of the diameter of the limb. This dressing was retained about three months, when the wire was removed, the union being established. In this case the apposition was perfect; the upper end of the lower fragment presented a superficial socket; while the lower end of the upper fragment was rounded to correspond with the cavity below, both ends being covered with articular cartilage.

Mr. C. fractured his thigh, obliquely, near the middle, on the fourth of July. On the following December there was an inch and a half of shortening, and no ossific union; the patient being unable to bear much weight on the limb, and obliged to use two crutches in walking. An attempt was made to break up the ligamentous union, bringing the limb to its proper length, and then to perform Brainard's operation. The limb was accordingly dressed as for recent fracture, and extension attempted. As much power was applied as the patient could bear, and continued forty-eight hours. At this time there was no appreciable lengthening of the limb, but great pain, throbbing, and soreness were the result of the extension. This plan was then given up, and, to take advantage of the inflammation thus excited, the starch bandage was applied. The patient was next detained in bed a few weeks, and then allowed to move about on his crutches a few weeks, the limb in the mean time getting more and more strength; so that, at the end of three months, he could walk with a cane, and is now perfectly cured, except the shortening.

Mrs. B., aged twenty-five, of good health and habits, fractured her humerus obliquely, and about the middle. The arm was well dressed and carefully attended; but just four months after, when Dr. Crosby first saw it, there was very free motion, and, of course, no union. Dr. Brainard's operation was performed on the 21st of November, 1856, and repeated on the twelfth day after; in four weeks union was complete.

*Ununited Fracture. Failure of two different Operations in succession.* PROF. M. GUNN, M.D. (Medical Independent, February, 1857, p. 326.)

"The patient, J. G., aged twenty-four years, was a man of fine physical development, and general appearance of health. Six weeks previously to Oct. 10th, 1856, he had sustained an oblique fracture of the humerus at its middle, the obliquity extending from behind and below, upward and forward. The arm was shortened about an inch and a half, the upper end of the lower fragment protruding under the skin at the outer border of the biceps muscle. In this position union had occurred, by a material, which, though it prevented restoration of the fragments to their original position, and the arm to its proper length, admitted freedom of motion in every direction.

"Treatment.—I adopted Professor Brainard's method, and pierced the extremities of the fragments and intervening tissue repeatedly, making but one puncture through the integument. The arm was then dressed with a roller and four splints. In addition, a firm body compress was placed between the arm and body, and a broad roller passed over all, including arm and body. The forearm was supported in a sling, and thus securely dressed, the result was awaited.

"Oct. 28th. As yet no appearance of increased action in the parts—operation repeated, and the arm redressed.

"Nov. 4th. There being still no signs of reparative effort, a seton was introduced between the fragments, and the arm dressed as before.

"Nov. 17th. Still no increase of action at seat of injury. All dressings were now removed, and the seton being still retained, the arm was left to swing for three or four days, when the dressings were re-applied.

"Nov. 20th. All means having thus far failed to excite the desired action, I resolved on exsection of the intervening tissue and the extremity of each fragment, so as to bring the fresh bone surfaces together. The seat of the fracture was identical with the spiral portion of the musculo-spiral nerve, and as its normal relations were necessarily altered by the mal-position of the lower fragment, I endeavored to modify the operation so as to avoid its injury. An incision was accordingly made, commencing at a point just anterior to the insertion of the deltoid, and extending downward along the outer border of the biceps, with a slight inclination outward. The bone was reached and its section commenced with a saw, and completed with bone forceps. No vessels requiring ligature, the wound was closed with the requisite sutures and dressed, and I congratulated myself on the completion of the operation without division of the nerve. But a paralysis, complete in the carpal and partial in the digital extensors, showed too plainly that the nerve had sustained injury, undoubtedly by the bone forceps in completing the section of the upper fragment. The arm was firmly secured in softened binder's board, the edges of which approached, but did not cover the wound, and the body dressings were applied.

"Nov. 28th. Removed sutures—union by first intention except at a small point in the centre of the incision. The wound healed from this time rapidly, and soon converted the case into simple fracture. Patient's health continued good, and I confidently expected a firm union.

"Jan. 9th, 1857. Fifty days after the operation the dressings were all removed, and not only no union had taken place, but there appeared not the slightest effort at repair."

*Dislocation of the Processes of the Cervical Vertebræ.* EDWIN R. MAXSON, M.D., of Geneva, N. Y. (Buffalo Med. Journ., Jan. 1857, p. 479.)—A girl, aged nine, while at play, on the morning of October 27th, 1856, by a sudden turn of the face toward the left shoulder, suffered a pain in the back of the neck, became faint, and found herself unable to turn the head back to its natural position. She was immediately placed in the most comfortable position, and recovered from the faintness somewhat, until she was moved to her bed at night, when she again became faint, restless, and irritable, resting but little during the night. No improvement, but rather more complaint of pain in the neck throughout the second day. Late in the evening of the second day, in attempting to move her again to her bed, they accidentally "turned the face a little more toward the left shoulder, as they supposed, when she was severely convulsed for a considerable time; after which she became very faint, and could not bear to be much raised in bed."

Dr. Maxson saw her at this time, and found her with the head still fixed in its position facing the left shoulder. The convulsions had subsided, but more or less spasm of the muscles continued to be aroused by any attempt to move her. Passing his hand along the spinous ridge of the neck, he discovered "an irregularity,—the spinous process of the fifth or sixth cervical vertebra appearing to the right of the one below." He was now confident "that there was displacement of the processes of the fifth or sixth cervical vertebra to such an extent as to slip the transverse or oblique process, or both, of one vertebra from the corresponding process of the vertebra below; and that the transverse or oblique process, or both, of one vertebra were caught against the corresponding process below; and hence the inability to turn the face from toward the left shoulder."

After stating to her parents the condition of the child, and the danger of an attempt to replace the parts, as well as the danger of delay, he proceeded, at their request, to the reduction, which was happily effected, and is thus described:

"I grasped the head with both hands, and proceeded according to Desault's method, only I first carried or turned the face very gently a little further toward the left shoulder, to (if possible) disengage the process; then, lifting or extending the head, I turned the face very gently toward the right shoulder, when the difficulty was at once overcome, and she exclaimed 'I can move my eyes.'"

Her countenance soon acquired a more natural appearance; the faintness passed off; she rested quietly through the night, and continued perfectly well.

*Complete Dislocation of the Cervical Vertebræ—Reduction on the tenth day. Recovery.* DANIEL AYRES, M.D., LL.D., Brooklyn, N. Y. (N. Y. Journal of Medicine, Jan. 1857, p. 9.)—A laboring man, thirty years of age, tall and muscular, but not fat, with a neck longer than the average among men of equal height.

"On the evening of the second of October he became intoxicated; was brought home insensible, and did not recover from the combined effects of the shock and his libations until the following morning, when he was supposed by his wife to be laboring under cold and a stiff neck."

Nothing was done until Dr. Ayres was asked to examine him on the ninth day after the accident.

"With some assistance and great personal effort, he was able to get out of bed, moving very slowly and cautiously. Desiring to expectorate, he was obliged to get down on his hands and knees, which he accomplished with the same deliberation. When seated in a chair, the head was thrown back and permanently fixed; the face turned upward with an anxious expression. The anterior portion of the neck, bulging forward, was strongly convex, rendering the larynx very prominent. The integuments of this region were exceedingly tense and intolerant of pressure. The posterior portion of the neck exhibited a sharp, sudden angle at the junction of the fifth and sixth cervical vertebræ, around which the integuments laid in folds. It was difficult to reach the bottom of this angle even with strong pressure of the fingers, and of course the regular line formed by the projecting spinous processes was abruptly lost. He complained of intense and constant pain at this point, which was neither relieved nor aggravated by pressure. With difficulty he swallowed small quantities of liquid, pausing after each effort, and could not be induced to take solid food, since the first attempt to do so after the accident was followed by violent paroxysms of coughing and choking. His breathing was obstructed and somewhat labored, being unable fully to clear the bronchia of their secretion. This, however, seemed rather an effect of the tense condition of the soft parts of the neck, than the result of pressure upon the spinal cord, since he presented no evidence of paralysis, either of motion or sensation, in parts below the neck. The sterno-cleido-mastoid muscles of both sides were felt quite soft and relaxed.

"But one conclusion could be formed upon this state of facts, to wit: that the oblique processes of both sides were completely dislocated. The marked rigidity of the head seemed to preclude the probability of fracture through the vertebral bodies, and although the cartilage might be separated anteriorly, yet, the body not pressing backward sufficiently to produce paralysis of the cord, it was hoped that the posterior vertebral ligament remained uninjured; it was, therefore, determined to make an effort at reduction on the following day.

"The patient was placed upon a strong table, in a recumbent position, with a pillow resting under the shoulders, the head being supported by the hand during the administration of chloroform, of which an ounce was given before anæsthesia ensued. Counter-extension being made by two folded sheets placed obliquely across the shoulders and properly held, the head was grasped by one hand placed under the chin, the other over the occiput, and by steadily and firmly drawing the head directly backward, and then upward, an attempt was made at reduction, but failed for want of sufficient power. Dr. Ingraham was then requested to place his hands immediately over my own in the same position as before, and steady traction was again made in the same direction. Our united strength was required in drawing the head backward and upward, to dislodge the superior oblique processes from their abnormal position. When this was felt to be yielding by Dr. Cullen, (who kept one hand constantly at the seat of dislocation,) Dr. Potter was directed to place his hands under our own, still in position, and assist in bringing the head forward; at the same time the chest was depressed toward the table. The bones were distinctly felt to slip into their places; the line of the spine was instantly restored, the head and neck assuming their natural position and aspect. As soon as the patient became conscious, he expressed himself ignorant of what had taken place, but free from pain, and, in his own language, 'all right.' A bandage was arranged to support the head and keep it bent forward. He had an anodyne for two nights following, after which no further treatment was necessary, and at the end of one week he had complete control over the movements of the head and neck. Beyond the debility and emaciation immediately dependent upon protracted fasting and loss of rest, he has experienced no uneasiness since the operation. His appetite is now good, and all the functions perform their duty normally. In a subsequent inquiry, to determine if possible the cause of the accident, he states that he distinctly recollects going into a store in Atlantic Street, near the ferry, and there having angry words with an acquaintance; that he left the store and was proceeding up the street, (which is here a rather steep ascent,) when he was violently struck from behind, over the lower portion of the neck. He likewise remembers falling forward and striking against some object, but does not know what it was, nor what took place until the following morning."

Two colored lithographs, representing the appearance before and after reduction, accompany this paper, which is further enriched with practical remarks, and with a review of leading authorities on the subject.

*Apparatus for the Reduction of Dislocations of the Fingers or Thumb.* RICHARD J. LEVIS, M. D., of Philadelphia. (Am. Jour. Med. Sci., Jan. 1857.) pp. 62, 63. With two wood-cut illustrations.

"It consists simply of a thin strip of any hard wood, about ten inches in length, and one inch, or rather more in width. One end of the piece is perforated with six or eight holes, arranged [as in the wood-cut] lengthwise in two parallel lines. The opposite end is partly cut away, forming a projecting pin, and leaving a shoulder on each side of it. Toward this end of the strip, a sort of handle-shape is given to it, so as to insure a secure grasp to the operator.

"Two pieces of strong tape, or other material, about one yard in length, are prepared. One of these is passed through the holes at the end of the strip, leaving a loop on one side. The other tape is passed through another pair of holes, according as it may be a thumb or finger, to which it is to be applied, or varied to suit the length of the finger, leaving a similar loop. If a dislocated thumb is to be acted on, the second tape should be passed through the holes nearest the first. The ends of each separate tape are then tied together.

"To apply the apparatus, the finger is passed through the loops. The loop nearest the first joint is then tightened by drawing on the tape, which is then brought along the strip to the opposite end, across one of the shoulders, and secured by winding it firmly around the projecting pin. The other tape is tightened in a like manner, crossing the other shoulder, and winding around the pin in an opposite direction, when, for security, the ends of the tapes are finally tied together.

"By this arrangement is gained a very simple means of making powerful extension; a leverage power by which the dislocated phalanx may be made to follow the rounded surface of the opposite articulation; and a power of rotating it while extension is being made, so as to turn one of the small condyles of the luxated phalanx at a time, under the unyielding lateral ligaments of the joint.

"If properly applied, without the slightest painful constriction of the finger or thumb, this apparatus is perfectly unyielding to any force applied in reduction, and it must break rather than slip from its hold.

"The control thus given to the operator, with its ready preparation at an emergency from materials everywhere at hand, give the apparatus decided advantages over the simple traction of the 'clove hitch,' or the more expensive and complicated devices which have been used for the purpose."

*Improved and Easy Method of Reducing the Backward Dislocation of the First Phalanx of the Thumb.* B. CUTTER, M.D., Woburn, Mass. (Boston Med. and Surg. Journ., Oct. 1, 1857, p. 172.)—After referring to the well-known difficulty in reducing this luxation by extension, and mentioning the various instruments and expedients for maintaining traction, Dr. Cutter proposes "a better way," which, for several years past, Prof. Dixi Crosby, of Hanover, N. H., has been accustomed to describe and recommend.

"Prof. C. remarks, in his lectures, that he has never failed in the few cases which have occurred in his practice, although in several instances all other methods usually adopted had been tried without effect; and, in his opinion, this method is equally applicable to similar dislocations of all the phalanges. I have verified this last opinion in one instance, the only one I have met with.

"The first case occurred about six years since; the subject, an Irish lad, about eight years old, with his *left-hand thumb* dislocated. After some ineffectual attempts to reduce it by his parents and neighbors, I was called. Having a general idea of Dr. Crosby's method, I proceeded to put it in practice. I placed the patient in a common chair, and took a seat in another at his side, both of us facing the same way. An assistant sat behind us, to hold the boy's elbow fixed. I then took hold of the metacarpal bone with my right hand, my forefinger passing between his thumb and forefinger, and my thumb resting on the top of the metacarpal bone, with its end touching the dislocated end of the phalanx. With my left hand I tipped the phalanx back until it stood perpendicularly on the metacarpal bone; then pressing the phalanx forward by the end of my right thumb, it was readily carried by flexion into place, and the joint restored to its natural condition. The manipulation was performed in a twentieth part of the time taken to describe it. A bandage was applied for a few days to prevent displacement, and some evaporating lotion to subdue inflammation.

"About the same time another similar accident occurred in an adult, an officer in the Customs, which in my absence was treated according to the same principle by my friend Dr. Rickard, and with equal success.

"The next case, a young girl about thirteen years of age, had the first phalanx of the *right* thumb dislocated on the back of the metacarpal bone. As she had suffered considerably in the attempts to reduce the dislocation, she and her friends insisted on using some anæsthetic, and, although I considered it unnecessary, she was gratified. As it was the right thumb that was injured, I seized it with my left hand, the forefinger underneath and my thumb on the back of the metacarpal bone. With my right hand I tilted up the phalanx until the end of the joint rested upon the metacarpus; then pressing it forward with the end ot my left thumb, I flexed the phalanx into the line and place with ease."

In a more recent publication of this paper, with additions, in the *Am. Journ. of Medical Sciences*, (April, 1858,) there is appended a note from Dr. Crosby, in which he states that he has practiced and taught this method since 1827. Dr. Cutter's paper is illustrated with marginal diagrams.

*Cases of Dislocation of the Femur reduced by Manipulation.*—CASE I. *On the Dorsum Ilii.* CHAS. H. BAKER, M.D., Genesee Co., N.Y. (Buffalo Med. Journ., March, 1857, p. 624.)—A lad, seven years of age; reduced at once, without anæsthesia, three hours after the accident.

CASE II. *On the Dorsum Ilii.* T. G. MCELBRIGHT, M.D., Nashville, Ohio. (Western Lancet, April, 1857, p. 255.)—A lad, aged seven years; anæsthesia produced with equal parts of ether and chloroform; reduction effected by Reid's method, within thirty seconds.

CASE III. *On the Dorsum Ilii.* C. E. ISAACS, M.D., Brooklyn. (N. Y. Journ. of Med., Nov. 1857, p. 418.)—A boy, aged eight years, injured two days previously; head of femur found to be in sciatic notch. Chloroform having been given, dislocation was restored in less than half a minute by manipulation according to Reid's method.

CASE IV. *On the Thyroid Foramen.* Professor C. S. BLACKMAN. Reported by N. J. SAWYIER, M.D. (Western Lancet, June, 1857, p. 419.)—J. T., aged twenty-seven, fell some thirty feet, striking with his left side on a plank floor. Reduction effected under chloroform, in eight or ten minutes, about twenty-four hours after the accident, by the usual process in Reid's method, except that the adduction across the sound limb was carried to a greater extent than in dislocation on the os ilii.

CASE V. *On the Thyroid Foramen.* R. T. BRODIE, M.D., Assistant Surgeon, U. S. A. (Charleston Med. Journ., Sept. 1857, p. 626.)—A boy, aged fifteen, dislocated his thigh in wrestling, an hour previously. Chloroform was administered by inhalation to the extent of half a fluidounce before he became insensible. Reduction was then effected at once by manipulation.

CASE VI. *Into the Sacro-Ischiatic Notch.* S. R. HENRY, M.D., of Burlington, Iowa. (N. A. Medico-Chir. Rev., Nov. 1857, p. 933.)—A very robust muscular man, aged forty. After administering chloroform, "we flexed the knee upon the thigh, and gradually elevated the latter upon the body, then bent it downward toward the other side, keeping up manipulation upon the trochanter. During its descent, the head of the bone was felt, and heard to move from the notch and take up its position on the dorsum ilii. After repeating this proceeding two or three times, it slipped into the acetabulum."

CASE VII. *Reduction by Manipulation of a Femoral Dislocation of six months' standing.* MARTIAL DUPIERRIS, M.D., Havana. (N. A. Med.-Chir. Rev., March, 1857, p. 29.)—A cooley, aged about sixteen, arrived from China in a feeble sickly condition, having been disabled throughout the voyage, by a fall which dislocated his hip before embarkation.

"The permanent contraction of the surrounding muscles, almost universally present under similar circumstances, was wanting in this case; as before stated, they were all in a flaccid condition, except the great gluteal, which was painful to the touch. The limb was somewhat more emaciated than its fellow of the opposite side, owing, doubtless, to the obstruction to the circulation and enervation produced by adhesions of the vessels and nerves in their unnatural situations. No anæsthesia was attempted on account of the feeble condition of the patient. Extension was tried without effect, except to create excruciating pain. The next day the reduction was undertaken by flexion, as the safer and at the same time more available procedure.

"The patient being placed upon his back, and the trunk of the body made steady by assistants, with the left hand I grasped the upper part of the leg, placed the right hand upon the head of the thigh-bone in the iliac fossa, and then proceeded to flex the leg upon the thigh, and the thigh upon the pelvis. By this movement the great gluteal muscle was relaxed, and the head of the bone advanced, while with the right hand I directed the latter toward the cotyloid cavity. As soon as I judged the head to be immediately above the centre of the socket, I extended the leg, the thigh remaining flexed at a right angle; and then using the limb as a lever, I rotated it from within outward, and at the same time extended it by making a movement of circumduction in a similar direction. When, by these procedures, the limb was brought near to its opposite fellow, a snap, audible to the assistants, and of a deeper character than is ordinarily observed in the reduction of recent dislocations, indicated the return of the bone to its natural position; a fact which was further substantiated by the establishment of the original length and form of the member and the subsidence of the pain."

In spite of precautionary measures, the displacement was reproduced on four successive days. After this, however, the head of the bone retained its place; passive motion was daily employed, and all suffering ceased. He gradually improved, until, in a few months, he was entirely restored. The paper concludes with some useful practical remarks upon the conditions recognized as favorable to the operation for reduction of old dislocations.

*Dislocation of the Femur on the Dorsum Ilii. Reduction by extension with pulleys, sixty-four days after the Accident.* THOS. CAMPBELL, of the N. O. School of Medicine. (N. O. Med. News and Hospital Gazette, Jan. 1, 1857, p. 656.)—W. L. D., aged thirty-four, stout and healthy, entered the Charity Hospital, November 10th, 1856, for an affection which Drs. E. Martin and Choppin recognized at once to be a dislocation of the femur upward on the dorsum ilii. A rude attempt at reduction had been made, under the patient's direction, at the time of the accident. A second attempt was made a few days later, but without success; after which he was treated eight weeks for fracture of the neck of the femur.

On the 14th of November, Drs. E. Martin and Choppin had the extending and contra-extending bands and pulleys applied in the usual way. Extension, slow and gradual but steady, was made, until the head of the bone approached the acetabulum, when Dr. Choppin rotated the limb inward, and the head of the bone slipped into the socket. The time consumed in effecting this result was about one hour and a half.

*Dislocation of the Head of the Tibia forward upon the Thigh-bone.* PROF. S. D. GROSS. (N. A. Med.-Chir. Rev., March, 1857, p. 298.)—A very large, fat woman, married, aged forty-eight years, fell, suddenly, with her whole weight upon her knee. Dr. G. saw her four hours after the accident.

"The knee, which was very painful, and a good deal swollen, especially on the inside, appeared to be unusually wide, from side to side, a circumstance which was partly due to the tumefaction of the soft parts. The leg was one inch and a half shorter than the opposite one, and in a straight line with the thigh. The patella had sunk behind the head of the tibia into a sort of hollow which gave to the front of the joint a sort of flattened appearance. Upon grasping the bone, however, with the thumb and fingers, it was easily drawn forward, leaving a remarkable vacuity behind, in consequence of its distance from the inferior extremity of the femur. The condyles of the thigh-bone lay in the popliteal space, posterior to the head of the tibia, where they formed a large prominence, more distinct on the inside than on the outside, and situated, as it were, in the upper and back part of the leg, the muscles of which were unusually tense. The head of the tibia lay in front of the condyles, where its outlines could be easily traced with the eye and finger. Above this bone, as already stated, was the patella, with its ligament, and the tendon of the extensor muscles, forming a broad, thick cord in front of the thigh-bone, from which it was removed more than two inches. The leg was easily drawn away from its fellow, but could not be carried inward, showing that there was extensive rupture of the internal lateral ligament. There was no contusion of the soft parts nor any discoloration of the integuments.

"Chloroform having been administered, a stout lac was applied to the upper part of the thigh, and confided to an assistant, to make the requisite contra-extension, while extension was made by another assistant grasping the foot, the limb being in an extended position. Placing, now, my left forearm behind the knee, and requesting the aids to pull gently and steadily, I suddenly, with my right hand, bent the leg backward, and thus in a few seconds effected the reduction; the bone slipped into its proper situation with a distinct 'snap.' The limb being placed in an easy position, cold cloths were applied to the knee, and a grain of morphia administered to allay pain and prevent spasm.

"No untoward symptoms appeared after reduction. The patient kept her bed for nearly a fortnight, and medicated lotions were applied, after the first twenty-four hours, to moderate and subdue inflammation. Purgation and light diet were also enjoined. In due time passive motion was instituted; the limb was frequently bandaged; and in less than a month from the accident, the woman was able to walk about the house with the aid of crutches. The joint, however, remained weak a long time, and even now, several years after the occurrence of the injury, the slightest fatigue is attended with temporary lameness."

*Paracentesis Thoracis.* H. I. BOWDITCH, M.D. (Boston Med. and Surg. Journ., June 4, 1857, p. 349.)—Dr. B. writes, in continuation of his articles published in the *Am. Journ. of Med. Sci.* (April, 1852,) and in the *Am. Med. Monthly*, (January, 1852):—

"Since my last article on thoracentesis, I have been more confirmed than ever in my belief of the importance of this operation as a remedial measure, to be used *not as a last resource*, but like any other simple remedy, if necessary, at any period of the disease. I still use the exploring trocar, although, in some instances, where there has been a tendency to a re-accumulation of fluid, I have used a larger instrument.

"Since that article (October, 1853) I have operated on thirty-seven persons, and have punctured sixty-one times, either with relief or great relief, in all but one. This person was very intemperate in her habits, and was stupid with liquor when I operated; but the dyspnœa was so very great as to threaten immediate death. She was relieved temporarily; but sank about twenty-four hours after the operation. With this exception, in not a single instance was there any untoward result."

He then gives the details of a few of the more interesting cases, and thus continues:—

"I have thus given you the more interesting of my recent cases. The notes are very brief. The records I have of them are ample. In a word, since April 17th, 1850, I have operated upon sixty-two individuals, of both sexes and all ages. I have punctured one hundred and eleven times. I know of nothing in practical medicine which has afforded me more satisfaction than this simple operation. I use designedly the expression—practical *medicine*, in contradistinction to surgery. The perfect simplicity of the operation, to one satisfied of the correctness of his diagnosis, allies it to venesection or vaccination. I am well aware that many will wonder, and some perhaps will scoff at this classification. To such I would say—Do not theorize on your fears; *try the operation*, and then you can judge more clearly. You will find that, as performed in these cases, (viz., with the exploring trocar,) it is, 1st, as a general rule, less painful than a blister; 2d, that (if I may judge from my cases) it *never* does harm; 3d, when fluid is obtained, it *always* gives relief, either temporary or permanent; 4th, that very often it is the chief, if not the sole means capable of relieving severe symptoms, and even of saving life.

"If these statements are true—and I am as convinced of their truth as I am of anything in my whole medical experience—I am justified in asserting, that a physician does wrong and acts foolishly who allows any patient to suffer months or years of misery, or even death itself, from pleuritic effusion, at any age—from any cause and with any complications—without at least *a trial of thoracentesis.* I write thus strongly because I fear that surgeons of even the highest reputation still shrink from performing this operation. This fear, I presume, is owing to their considering it as similar to the operation laid down in all, or almost all, of their own manuals. From that operation they ought in most cases to shrink. That which is here indicated is of a totally different character, and is, so far as my experience goes, harmless."

*Lithotrity.* Dr. C. S. FENNER, of Memphis, Tenn. (New Orleans Medical and

Surgical Journal, vol. xiii. No. iv., p. 448.)—A case of stone in the bladder is here reported "simply as an instance of the facility with which, in favorable cases, small stones may be broken and passed off by piecemeal, and the necessity for a painful and dangerous cutting operation obviated." A negro, about thirty years old, unusually intelligent, and, except a valvular disease of the heart, of good general health, had suffered with symptoms of stone for ten years; he was at times incapacitated for field-labor, and had repeatedly solicited his master to have him cut.

The stone was seized, and broken into fragments, with a medium-sized lithotriptor of Heurteloup with rack movement, and two or three of the larger pieces were then crushed. The whole operation did not last five minutes; blood was lost, and the pain felt was trifling. After having passed fragments of stone for two days he felt entirely relieved, and the sound gave no indication of any foreign body in the bladder. He slept without waking throughout the third night, which he had not done for years. On the third day he went to the field and performed his accustomed amount of labor. From that time, "early in November," until his death, which happened "late in the spring from a disease of the heart," he had no symptoms of the stone.

*Extraction of a piece of Lead-pencil from the Bladder by a small Incision through the left side of the Perinæum.* S. D. GROSS, M.D., Prof., &c.—A farmer, aged thirty-nine, while introducing into his urethra, according to his habit, in order to facilitate the flow of urine, a piece of common lead-pencil, allowed it to slip into the bladder. The usual irritation followed, and some twenty-six days subsequently he presented himself to Dr. Gross, in Philadelphia, having travelled from his home in Wellington County, Canada West. The pencil was readily detected with the sound. A cathartic was ordered, and the next day an unsuccessful attempt was made to extract the foreign body through the urethra.

The lateral operation of lithotomy having been determined on, it was performed, on the morning of the third day after his arrival,—

"In the usual manner, except that the incisions were very small; the pencil was found to be behind the pubic symphysis, and was removed without the slightest difficulty. Very little blood was lost. Urine had ceased to flow from the wound on the sixth day. Some febrile irritation occurred at the end of the first week, but soon disappeared under soothing treatment. He left for home on the nineteenth day after the operation."

The wound was nearly cicatrized, and all the urine came away through the natural passage. The vesical distress had subsided, and there was but little pain in urinating. The urine was sometimes high colored, and contained a small quantity of sabulous matter. In all other respects the patient was entirely well. The pencil, which was slit in half, the part containing the lead having gotten into the bladder, was two inches and five-eighths in length and was conical at one extremity. It was coated with a deposit which weighed about one drachm, and consisted of a small quantity of uric acid, the rest being triple phosphate.

*Case of Lithotomy for numerous calculi, with a sequestrum of bone in the neck of the bladder. Cured.* D. B. VAN TUYL. (Medical Independent, Nov. 1857.)

The operator reports this case as an instructive and striking instance of the fact, long since demonstrated, that "excessive and long-continued exercise of any particular set of muscles will cause inflammation of the bony tissue to which such muscles have their attachments; and that such inflammation may result in hypertrophy, caries, or necrosis, with a train of concomitant ills such as human flesh is seldom heir to."

The important points in the history of the case are sufficiently apparent in the writer's summary, which is here appended:—

"Here we have the case of a man who, from his own account, has suffered with the worst forms of rheumatism for more than twenty years. But, *in reality*, it is not probable that he has ever been affected with that disease in the slightest degree.

"Mr. L. is a man of five feet and six inches in height, with an average weight of only one hundred and twenty-eight pounds; but he is a man of great muscular strength. When he was twenty years of age, he supposed he was the strongest man, *of his weight*, in New England. His muscular power, therefore, was equal to the task of running five miles, or perhaps a much greater distance. But the bones which were required to sustain the shock of the repeated contractions of those muscles which are most powerfully exercised in running were not equal to their part of the performance. The parts to which those muscles were attached became inflamed to a degree which gave the disease the *appearance* of rheumatism; and what the result would have been had he not exposed himself to the fatigue of that twenty mile walk can only be conjectured. But that additional irritation hastened the work of destruction. The inflammation, which had been of a subacute character, at once became active and violent, and exfoliation of the anterior processes of both ossa ilii, also a part of the tuberosity and ramus of the left ischium, and a part of the descending ramus of the left pubic bone, was the result.

"The sores on the lower part of the abdomen, as we learn, continued to discharge, one three, the other thirteen years; but as soon as the last piece of bone was extruded, the ulcer healed. The cicatrices from those sores extend, in a crescentic shape, from the anterior superior spinous process of one ilium to that of the other. Had the real cause of these abscesses been suspected, all his sufferings in *those parts* might have been terminated at any time, simply by enlarging those sinuses and removing the exfoliations.

"But when nature, after a most protracted, painful, and unassisted labor, had brought forth a whole litter of those sequestra, *one* still remained, and that in a position which bid defiance to her best-directed efforts for its removal. This was on the internal and posterior part of the descending ramus of the left pubic bone. The exfoliations which had escaped through the opening in the thigh had doubtless come from the immediate neighborhood of this remaining one, a little lower down. But when this piece had ulcerated through the parts which covered it, it found itself, as it were, *looking into the neck of the bladder;* and had it become fully detached, would have fallen into that viscus. But remaining *in situ*, and being constantly in contact with the urine, it became a nucleus, or surface, upon which was deposited the numerous small calculi and fragments which were removed during the operation. The position of the bone was such that, in passing a catheter, (an operation which Mr. L. had performed upon himself frequently for many years,) the point of the instrument glided lengthwise along the bone; and whenever the deposit had become so great as to prove an obstruction to the free passage of the catheter, the instrument would detach the deposit, which would fall into the bladder, and there become a nucleus for still further deposit. This accounts for the great number and variety, in size and shape, of the calculi; some that had recently been detached being still perfect casts of that part of the bone upon which they had been deposited; while others, which had served for years as nuclei, had attained to considerable size.

"There were about twenty of those pieces, from the size of a pea to that of a filbert, besides a great number of smaller fragments, and a small spoonful of sandy matter."

The patient was walking about the streets in less than three weeks after the operation; and for more than a year has been engaged in his ordinary business, travelling, &c., in perfect health.

*A Statistical Report of Forty-six Operations for Stone in the Bladder, with the best method of performing Lithotomy.* PAUL F. EVE, M.D. (Nashville Jour. of Med. and Surgery, Aug. 1857, p. 81.) The first part of this paper is occupied with a synopsis of twenty-one cases, in continuation of a report of twenty-five similar cases which was published in the April number of the Am. Jour. of Med. Sciences for 1852.

"Since October, 1841, the date of my first operation, a period of a little over fifteen years, I have operated on forty-six cases of urinary calculi, thirty-eight of them under chloroform, without an unpleasant result from it, and have removed one hundred and sixty-six stones. The following tables will present the statistics of these cases in regard to the date, age, sex, race, residence, method of operation, number of calculi, and the result of each one.

*Statistics of Forty-six Operations for Urinary Calculus.*

| No. | Date. | Age. | Sex. | Race. | State. | Operation. | No. of Calculi. | Result. |
|---|---|---|---|---|---|---|---|---|
| 1 | 1841 | 8 | Male. | Mulatto. | Ga. | Bi-lateral. | 1 | Speedy Recovery. |
| 2 | 1843 | 6 | " | White. | Ga. | " | 1 | " " |
| 3 | 1843 | 3 | " | Mulatto. | Ga. | " | 1 | " " |
| 4 | 1845 | 3 | " | White. | Ga. | " | 1 | " " |
| 5 | 1845 | 34 | " | " | Ga. | Lithotrity. | 1 | " " |
| 6 | 1846 | 24 | Female. | Black. | Ala. | Vaginal Section. | 1 | " " |
| 7 | 1847 | 20 | Male. | White. | Ga. | Bi-lat·ral. | 1 | " " |
| 8 | 1847 | 20 | " | " | Ga. | " | 1 | " " |
| 9 | 1848 | 5 | " | " | S. C. | " | 1 | " " |
| 10 | 1848 | 5½ | " | " | S. C. | " | 1 | " " |
| 11 | 1849 | 50 | " | " | S. C. | " | 117 | " " |
| 12 | 1849 | 6 | " | Black. | S. C. | " | 1 | " " |
| 13 | 1849 | 4 | " | White. | Ga. | " | 1 | " " |
| 14 | 1849 | 10 | " | " | Ga. | " | 1 | Died of Dysentery when wound [nearly healed.] |
| 15 | 1850 | 12 | " | " | Ga. | " | None. | Speedy Recovery. |
| 16 | 1850 | 7 | " | " | Ga. | " | 1 | " " |
| 17 | 1851 | 4 | " | " | S. C. | " | 1 | " " |
| 18 | 1851 | 7 | " | " | Ga. | " | 1 | " " |
| 19 | 1851 | 7 | " | " | Ga. | " | 2 | " " |
| 20 | 1851 | 77 | " | " | Ga. | " | 3 | Death in sixty hours. |
| 21 | 1851 | 24 | " | " | Tenn. | " | 1 | Speedy Recovery. |
| 22 | 1851 | 5 | Female. | " | Ky. | " | 1 | " " |
| 23 | 1852 | 7 | Male. | " | Tenn. | " | 1 | " " |
| 24 | 1852 | 24 | " | " | Tenn. | " | 1 | Death sixth day. |
| 25 | 1852 | 65 | " | " | Tenn. | " | 1 | Death about thirteenth day. |
| 26 | 1853 | 3 | " | " | Tenn. | " | 1 | Speedy Recovery. |
| 27 | 1853 | 4 | " | " | Tenn. | " | 1 | " " |
| 28 | 1853 | 10 | " | " | Ga. | " | 1 | " |
| 29 | 1853 | 20 | " | " | N. York. | Lithotrity. | 1 | " " |
| 30 | 1854 | 34 | " | " | Ga. | Bi-lateral. | 2 | " |
| 31 | 1854 | 12 | " | " | Tenn. | " | 1 | Speedy " |
| 32 | 1854 | 7 | " | " | Tenn. | " | 1 | " " |
| 33 | 1854 | 23 | " | " | Ga. | " | 1 | " " |
| 34 | 1854 | 26 | " | " | Ga. | " | 1 | " |
| 35 | 1855 | 5 | " | " | Tenn. | Lateral. | 1 | Speedy Recovery. |
| 36 | 1855 | 16 | " | Mulatto. | S. C. | Bi-lateral. | 1 | " " |
| 37 | 1855 | 9 | " | White. | Tenn. | " | 1 | " " |
| 38 | 1855 | 66 | " | " | Miss. | High Operation. | 1 | " " |
| 39 | 1856 | 2½ | " | " | Tenn. | Bi-lateral. | 2 | " " |
| 40 | 1856 | 11 | " | " | Tenn. | " | 1 | " " |
| 41 | 1857 | 26 | " | " | Tenn. | Dilatation. | 1 | " " |
| 42 | 1857 | 22 | " | " | Tenn. | Bi-lateral. | 1 | Slow Recovery. |
| 43 | 1857 | 19 | " | " | Tenn. | " | 1 | Speedy Recovery. |
| 44 | 1857 | 8 | " | Black. | Tenn. | " | 1 | " " |
| 45 | 1857 | 61 | " | White. | Tenn. | " | 1 | " " |
| 46 | 1857 | 9 | " | " | Tenn. | " | 1 | " " |

"Rejecting the case of death from prevalent dysentery when the wound was nearly healed, the mortality exhibited by these statistics is 1 in 15⅓, and 35 of 46 had a speedy recovery."

*Summary of the Forty-six Cases.*

| NO. OF CASES. | AGE. | SEX. | RACE. | OPERATION. | NO. OF STONES. | RESULT. |
|---|---|---|---|---|---|---|
| | 27 under 15.<br>15 adults.<br>1 aged 61.<br>1 " 65.<br>1 " 66.<br>1 " 77. | 44 males.<br>2 females. | 40 whites.<br>3 mulattoes.<br>3 blacks.* | 40 bi-lateral.<br>1 lateral.<br>1 vaginal.<br>1 high.<br>1 dilatation.<br>2 lithotrity. | 117 in one.<br>3 in one.<br>6 in three,<br>2 each.<br>† 40 in 41. | Death occurred from dysentery in one of the 27 under 15.<br>Death in one of the 15 adults.<br>Death in 2 of aged. 36 had a speedy recovery. |
| 46 | 46 | 46 | 46 | 46 | 166 | |

Dr. Eve informs us that he "had not declined to operate in a single case under his care where, in the opinion of others, it ought to have been done. In one instance, a patient died a few days after being placed on the table, though no operation was attempted, and two others expired after leaving home to seek relief. I know now that I operated on two cases which were at the time beyond all expectation of a cure, and another one, it is possible, might have been relieved by lithotrity or the high operation.

"As to the best method of performing lithotomy, it is very certain, as in many other operations, that no one in particular should be adopted to the exclusion of all others. Each case is a problem to be solved best by a careful study, and the adaptation of therapeutic means to meet all its peculiarities. While perfection may not be attained by any plan yet proposed for removing stone from the bladder, that operation attended with the least risk to the patient and followed most generally with success, is the one to be selected.

"I acknowledge, in the child or boy up to about fifteen years of age, that an experienced surgeon may, with the scalpel alone, make a capital operation and obtain good results; but in a majority of cases and under ordinary circumstances, I have little doubt, when the method about to be described is understood, it will be admitted to be the best. My staff has, at the upper part of its groove, an opening large enough to admit the beak of the lithotome, and which is there so contracted as to prevent the escape of the cutting instrument until it arrives near the extremity, and has consequently entered the bladder. It acts, in other words, as a safe and certain director to the lithotome. It guides it with unerring precision into the bladder, and thus prevents the rectum being wounded, or the cutting instrument passing into the space between these two organs,—an event which has too often happened.

"I have also added a slight modification to the double lithotome cachée. I found the shoulder of it too large, especially for children, to enter freely upon the staff when in the urethra. To prevent laceration of this canal I have had two small blades put upon the shoulders, so that the instrument may cut its way into the bladder.

"In operating for urinary calculus, I make an inverted ⅄ incision in the perineum; thus, with a scalpel, beginning at the bulb of the urethra, an incision three-quarters of an inch in length is made to the median line, then the knife turned to the left, to terminate about midway between the anus and tuberosity

* I have had only one colored patient of the West.

† In one case I failed to remove the stone. This was in 1850, and two months ago the patient arrived here, seeking relief from symptoms of urinary calculus, under which he has labored ever since I operated upon him. He is a member of the class in the Literary Department of our University.

of the ischium. A similar leg to the inverted ⅄ is then made on the other side, but with its cutting edge turned up, and arriving at the median line, the point of the scalpel is entered down to the staff in the urethra, upon which the lithotome is now conducted into the bladder. The staff being removed, and the half-turn given to the lithotome, the blades are expanded and the bi-lateral section made in withdrawing this instrument, which will be found to correspond very nearly with the external inverted ⅄ incision previously made in the perineum.

"The advantage of this external incision over the crescentic one of Dupuytren is the facility with which it may be made and the urethra opened upon the staff. I make as small an opening in the prostate gland as will permit the extraction of the stone, knowing from experience the parts may be considerably dilated by gentle persevering traction with the forceps grasping the calculus. And the only unpleasant occurrence ever met with in the treatment of cases thus operated upon, placed under my care, has been hemorrhage; and how this may be arrested I have described. The development of the vessels about the neck of the bladder and perineum in calculous patients, and the irritation created by the passage of urine over the wounded surface, will account for the special and unavoidable liability to bleeding after lithotomy."

*Instrument for the Radical Treatment of Reducible Inguinal Hernia.* J. W. RIGGS, of Plainfield, N. J. (New York Journ. of Med., July, 1857, p. 143.)—Dr. Riggs is here announced to have "devised an instrument for the introduction of a seton into the inguinal canal, where there is a reducible inguinal hernia, for the purpose of exciting a sufficient degree of inflammation to occlude this passage; the instrument consists of a long curved canula, containing a trocar, having an eye near its pointed extremity. To operate, the hernia must be entirely reduced, and the canula passed up, upon the finger, to the internal ring; the trocar is then thrust upward through the integument, and a seton being passed through the eye, it is withdrawn into the canula, and both are withdrawn from the canal, thus leaving the seton lying in the inguinal canal. The seton may or may not be medicated. This operation has been performed several times with complete success."

A paper, "*On the Radical Treatment of Reducible Inguinal Hernia, with the Description of a New Instrument and a Report of Successful Cases,*" has been published by Dr. Riggs (now of New York) in the New York Journal of Med. for March, 1858. This article contains a full and detailed description of the inventor's mode of operating, and a wood-engraving of his instrument, which, at the request of the senior editor of the Review, are introduced into this Report, in order to facilitate the comprehension of the mode of operating above referred to, as well as to afford a better idea of the means employed.

"*Operation.*—Before proceeding in the operation itself, the surgeon will, of course, provide himself with whatever substance is to be drawn into the canal, whether this consist of a small skein of silk, or compressed sponge; the latter, in our estimation, being entitled to the preference.

"The patient, placed upon his back, with the *hips somewhat elevated,* (a good precaution, particularly when the openings are large, and the viscera liable to escape, while in the horizontal position,) the surgeon, standing or sitting at the right side of the patient, after reducing the hernia, places the index finger of the left hand upon the integuments of the scrotum, anteriorly and at a point *not higher* than the juncture of the lower with the middle third of the pouch. Sufficient pressure being now made with the finger to catch and hold, upon its end, the tegumentary tissues of the scrotum; these are now to be carried, upon the end of the finger, upward over the testis and arch of the pubes, until, immediately above the bone, the abdominal ring is easily found, and into which the end of the finger readily becomes fixed; where, as a guide to the instrument, it is to remain stationary, until the bulbous extremity of the canula is made to take its place *fairly* and *securely within the external ring.* The instrument, in the right hand of the operator, and held at the serrated portion of its handle, between the thumb and fingers, something after the manner of holding a pen, is passed into the pouch of the invaginated scrotum, and made to glide along and upon the back of the finger to its destination within the ring. The finger may then be withdrawn, and the left hand being now liberated, the thumb of this hand may be placed at a point opposite the internal ring; where, by pressure, all danger from any tendency there may be to partial protrusion of the intestine through the internal ring can be effectually obviated, while, at the same time, the pressure thus exerted at this point tends materially to facilitate the passage of the instrument through the tissues. The instrument is now carried forward until the bulb approaches as nearly as practicable to the internal ring, when the handle is depressed upon the pubes, which serves to elevate its bulbous extremity, causing a prominence upon the surface, and indicating both to the touch and to the eye the exact point of its exit through the integuments. The operator now places the index finger of the right hand through the ring of the stylet, and with a single movement of the finger thus placed, pierces all the tissues involved in the operation, and brings the point and eye of the instrument into view upon the surface opposite the internal abdominal ring.

"The surgeon, or his assistant, now arms the stylet, by passing through its eye, for an inch or more, (as seen in the plate,) the free ends of a slender cord, or tractor, previously passed through the silk or sponge; when, by a single reversed or backward movement of the finger, which is still in the ring of the stylet, the instrument is entirely disengaged and freed from the tissues, being still, however, concealed from view within the pouch of the invaginated integuments. The entire removal now of the instrument leaves the free ends of the tractor passing through the puncture in the scrotum, and hanging loose below. These are now seized, and by the necessary traction the foreign body is drawn from above into the passage, to the distance of some two inches or more, and leaving its upper extremity protruding from the puncture above; when, dropping from his grasp one of the ends of the cord, the surgeon, by means of the other end, draws it entirely out, and thus completes this bloodless and comparatively painless procedure.

"*Dressing, treatment*, &c.—In ordinary cases, where the apertures are not very much distended, and in most cases, especially when the sponge has been employed, there are, at first, few indications for interference—none, indeed, beyond the necessary precautions to prevent the escape of the viscera; and, as before stated, when the compressed sponge is introduced, the secretions absorbed by this substance soon expand it so as, with the swelling and increased rigidity of the tissues which necessarily ensue, to effectually prevent the occurrence of the event alluded to. Independently, however, of the foregoing consideration, a given amount of equable and uniform pressure is requisite, in order to effect the occlusion of the passage, by means of the adhesive inflammation, to produce which the operation is designed. For this purpose, I am aware of nothing better than the introduction, under a bandage firmly drawn around the pelvis, of a large sponge previously dipped in water. This retains moisture longer than the cloth compress, does not so effectually exclude the air, and thus hinder or prevent the cooling process of evaporation from the inflamed part, while the pressure exerted by it is more equable and agreeable to the patient, and it thus fulfils at once, and in a very satisfactory manner, several important indications in the treatment.

"I am aware that the foregoing suggestions, relative to the treatment *subsequent* to the operation, may be regarded by the professional reader as supererogatory or arrogant, and I have been induced to offer them only from a consciousness of having witnessed very many instances of failure to cure hernia simply from too great and too long-continued pressure during the inflammation; the inevitable effect being to prevent that free and plentiful deposit of plastic lymph, on which the permanent closure of the hernial apertures and consequent cure of the malady is almost entirely, if not wholly dependent. These disastrous results have been repeatedly seen by the writer, both in cases where the cure was attempted by injection of the sac, and also when the requisite inflammation had been induced by the *truss*, and resulting in *apparent* cure; but when, by the continued and persistent application of undue pressure, absorption of the new deposits, (which might be expected,) and consequent destruction of the delicate and tender adhesions, and reopening of the hernial passage, have resulted from the unintelligent use of the very means intended for the cure of the disease."

Two cases, recently treated, of his own, are reported by Dr. Riggs, and six others, likewise recent in date of trial, in which the operation and treatment were conducted by Dr. Carnochan. In regard to these cases Dr. Riggs remarks:—

"The reader will have noticed that, in the eight operations above given, were comprised persons of various ages, ranging from sixteen to sixty-three years. There are also found, among these, ruptures of all dimensions, and, in point of duration, varying from one to twenty-six years. Some of these were under treatment for other diseases, and *none* of them can be regarded as the most favorable subjects for any kind of surgical operation.

"Notwithstanding the fact that among these was not *one* which would have been *chosen* for an untried operation, yet it is seen by the report, and is well worthy of mention, that in no instance was there sufficient constitutional disturbance scarcely to attract attention. The operation itself is very simple, and equally safe; is attended with no inconvenience to the operator, and to the patient with comparatively no pain."

## Figures and Tables

**Figure f1:**